# Prognostic Factors in Patients With Pulmonary Hypertension—A Nationwide Cohort Study

**DOI:** 10.1161/JAHA.116.003579

**Published:** 2016-08-29

**Authors:** Wei‐Ting Chang, Shih‐Feng Weng, Chih‐Hsin Hsu, Jhih‐Yuan Shih, Jhi‐Joung Wang, Chun‐Ying Wu, Zhih‐Cherng Chen

**Affiliations:** ^1^ Department of CardiologyChi Mei Medical CenterTainanTaiwan; ^2^ Department of Medical ResearchChi Mei Medical CenterTainanTaiwan; ^3^ Department of BiotechnologySouthern Taiwan University of Science and TechnologyTainan 710Taiwan; ^4^ Department of Healthcare Administration and Medical InformaticsKaohsiung Medical UniversityKaohsiungTaiwan; ^5^ Department of Internal MedicineCheng Kung University HospitalTainanTaiwan; ^6^ Division of GastroenterologyTaichung Veterans General HospitalTaichungTaiwan; ^7^ Department of PharmacyChia Nan University of Pharmacy and ScienceTainan CityTaiwan

**Keywords:** age, mortality, pulmonary hypertension, sex, Pulmonary Hypertension, Women, Risk Factors

## Abstract

**Background:**

Pulmonary hypertension (PH) is a rare but fatal condition. Large‐scale studies to examine the prognostic factors are lacking. In the present study, we aimed to investigate the factors associated with overall mortality in PH patients.

**Methods and Results:**

Based on Taiwan's National Health Insurance Database, we identified 1092 newly identified PH patients between 1999 and 2011. These patients were matched with 8736 healthy subjects based on propensity score calculated with age, sex, and chronic cardiovascular risk factors. Overall mortality, death incidence rate ratio, and hazard ratio were calculated. Patients with PH had a higher mortality than controls (56.45 versus 18.51 per 1000 person‐years, *P*<0.0001), with hazard ratio at 3.3 (95% CI: 2.92–3.73, *P*<0.001). The long‐term survival rates of the PH patients at 1, 5, and 10 years were 87.9%, 72.5%, and 62.6%, respectively, which were significantly lower than controls with 98.4%, 90.8%, and 83.6% at 1, 5, and 10 years, respectively. Among patients with PH, the mortality rate was higher in the older and male patients. However, after stratifying by age and sex, the younger (<50 years) and female patients had a higher risk. Regarding different etiologies of PH, chronic obstructive pulmonary disease and pulmonary embolism led to most cases of mortality (adjusted hazard ratio: 3.2, 95% CI: 2.76–3.71 and 4.64, 95% CI: 2.74–7.87, *P*<0.05).

**Conclusions:**

PH has high mortality, especially in females, and patients with younger age and with chronic diseases. Chronic obstructive pulmonary disease and pulmonary embolism contributed to an increased risk of mortality in PH patients.

## Introduction

Pulmonary hypertension (PH) is a rare but fatal condition.^1^ With the increase in research into the diagnosis and treatment of PH, it is important to provide updated information on the impact of this disease on mortality.^2^ In addition, it has been reported that the epidemiologic characteristics vary among different ethnicities.^2^, ^3^, ^4^ The Morbidity and Mortality Weekly Report Surveillance Summary reported that the age‐standardized mortality rates associated with PH range from 4.5% to 7.3% in African Americans and 5% to 5.5% in whites.^2^ In addition, the survival rates of PH at 1, 3, and 5 years have been reported to be 68%, 48%, and 34%, respectively, with an estimated median survival of 2.8 years.^5^ Given the large population in Asia, the potential prevalence of PH may be higher; however, large‐scale epidemiologic studies are lacking. In China, it has been reported that 6.6% of all inpatients have PH,^6^ with 65.9% of cases originating from congenital heart diseases (CHDs), 22.6% from left‐sided heart diseases, 5.7% from thrombotic diseases, 0.9% from respiratory diseases, and 0.6% from connective tissue diseases (CTDs).^6^ Another study reported that after 40.1 months of follow‐up, the survival rates at 1, 2, 3, and 5 years were 68.0%, 56.9%, 38.9%, and 20.8%, respectively.^7^ However, when these studies were conducted there was a lack of effective treatment, and with the improvements in updated treatment strategies the characteristics of PH may have changed.

Previous studies have reported that male sex and old age contribute to higher mortality;^8^ however, a recent registry reported an increasing trend of PH‐induced mortality and hospitalization in females.^9^ In addition, women were reported to account for 61% of all PH hospitalizations in 2002 and up to 63% in 2010.^2^ Thus, the impact of sex in PH‐associated survival remains controversial. Chakinala and colleagues reported that compared with congenital and idiopathic PH, systemic sclerosis and human immunodeficiency virus–induced PH led to worse survival.^10^ However, comprehensive and large‐scale studies on the outcomes of different etiologies of PH are lacking. Therefore, we conducted this nationwide study to investigate the risk factors associated with mortality in a cohort of patients with PH.

## Methods

### Data Source

Taiwan launched a single‐payer National Health Insurance (NHI) program on March 1, 1995. The NHI database provides data for nearly all the residents in Taiwan (coverage rate over 98% in 2009), making it one of world's largest and most complete population‐based data sets. The data used in this study came from the Longitudinal Health Insurance Database 2000, a subset of the NHI database containing all claims data from 1996 to 2011 for 1 million beneficiaries randomly selected in 2000. There were no significant differences in age, sex, and healthcare costs between the sample group and all enrollees at that time. The Longitudinal Health Insurance Database 2000 provides encrypted patient identification numbers, sex, date of birth, dates of admission and discharge, the International Classification of Diseases, Ninth Revision, Clinical Modification (ICD‐9‐CM) codes of diagnoses and procedures, details of prescriptions, registry in the Catastrophic Illness Patient Database, and costs covered and paid for by the NHI. The National Health Insurance Research Database has been described in detail in previous studies.^11^, ^12^ The accuracy of diagnosis of major diseases in the National Health Insurance Research Database, such as stroke and acute coronary syndrome, has been validated.^13^ This study was exempt from full review by the Institutional Review Board of Chi‐Mei Hospital (TCHIRB‐1030603‐W) because the data set comprised de‐identified secondary data.

### Study Design

This nationwide, population‐based, observational, retrospective cohort study was conducted to determine the association between PH and subsequent mortality. Two cohorts were enrolled in this study. The PH cohort, extracted from the entire original NHI database, consisted of patients with a first‐time discharge diagnosis of PH (ICD‐9‐CM codes 416.0 and 416.8) from January 1999 to December 2011. These codes were considered to be reliable for a diagnosis of PH on the basis of the clinical symptoms and a pulmonary artery pressure above 25 mm Hg. The control cohort (n=8736; 8 control patients for every PH patient) consisted of randomly selected patients who were not diagnosed with PH. The controls were matched using propensity score matching by age, sex, and chronic cardiovascular risk factors. We used propensity score matching to reduce selection bias because it can group many confounding covariates that may be present in an observational study. Propensity scores were computed by modeling a logistic regression model with the dependent variable as the odds of diagnosis of PH and the independent variables at baseline covariates (ie, age and sex) and selected comorbidities (Hypertension, Diabetes, Hyperlipidemia, and coronary artery disease [CAD] in our study). Thereafter, a Statistical Analysis System (SAS) matching macro “%OneToManyMTCH” proposed in the proceedings of the 29th SAS Users Group International was used in this study to match the propensity score.^14^ It allows propensity score matching from 1‐to‐1 to 1‐to‐N based on specification from the user. The macro makes “best” matches first and “next best” matches next in a hierarchical sequence until no more matches can be made. Each control subject is selected at the most once. The index date for each PH patient was the date of the initial diagnosis, and the date of the PH patient's index date was used to determine the index date of each matched control subject.

Age was classified into 3 categories: <50, 50 to 64, and ≥60 years. Concomitant chronic diseases included hypertension (ICD‐9‐CM codes 401–405, A260, A269, 4372), diabetes mellitus (ICD‐9‐CM codes 250, A181, A189, A229, A239, 3572, 3620), hyperlipidemia (ICD‐9‐CM code 272), and coronary artery disease (CAD; ICD‐9‐CM codes 410–414). In addition, we categorized patients according to the etiology contributing to PH, including CTDs (ICD‐9‐CM codes 701.1, 710.0, 710.2, 710.9, 714), CHD (ICD‐9‐CM codes 745.0, 45.11, 745.2, 745.1, 745.12, 745.3, 746.1, 746.7, 747.41, 745.4, 745.5, 747.0, 746.02, 745.60, 745.6, 746.2, 746.3, 746.82, 747.1), pulmonary embolism (ICD‐9‐CM code 415.1), idiopathic pulmonary hypertension (ICD‐9‐CM code 416), and chronic obstructive pulmonary disease (COPD; ICD‐9‐CM codes 490–496). To ensure that the selected cases had a primary episode of PH and to avoid potential confounding introduced by repetitive occlusions, patients with PH in an ambulatory setting before January 1999 were excluded.

### Outcomes

The primary outcome was mortality, and the secondary outcome was hospitalization within 30 days after the diagnosis of PH. Admissions for patients readmitted less than 1 day after discharge at the same or different hospitals were regarded as admissions for the same episode. Mortality was identified using the “in‐hospital death” code at discharge. However, many Taiwanese choose to “die at home,” and therefore the in‐hospital death code would underestimate the true hospital mortality rate. As enrollment in the NHI program is mandatory for everyone in Taiwan, and as registration must be withdrawn within 30 days after death, the subjects who were withdrawn from the NHI program within 30 days after discharge from the last hospitalization were presumed to have died. Repeated hospitalizations were defined as hospitalizations within 30 days after a diagnosis of PH. All subjects were followed up until death, being lost to follow‐up, or December 31, 2011, whichever came first.

### Statistical Analysis

The Student *t* test was used for continuous variables and the χ^2^ test for categorical variables. Incidence rates were calculated as the number of outcomes divided by the total number of person‐years of follow‐up. The risk of mortality was compared between the PH and control groups using the incidence rate ratio (IRR), as calculated by Poisson regression analysis. Cox proportional hazard regression analysis was used to calculate the adjusted hazard ratio (HR) for mortality. Kaplan–Meier analysis was used to calculate the survival rates, and differences in survival curves were analyzed using the log‐rank test. Since the pathogenesis may vary according to the etiology of PH, we examined whether the effect of PH on mortality was modified by the etiology, including autoimmune disease, CHD, pulmonary embolism, and COPD. For the secondary outcome (hospitalization within 30 days after a diagnosis of PH), multiple logistic regression was used to estimate the odds ratio among those with PH (others as the reference group). A 2‐tailed *P*‐value less than 0.05 was considered to be statistically significant. All analyses were performed with SAS software version 9.4 (SAS Institute, Cary, NC).

### Validation of the Accuracy of PH Diagnosis

To validate the accuracy of the PH diagnosis, we introduced 2 methods. First, we strictly redefined the diagnosis of PH to patients who not only fulfilled ICD‐9‐CM codes (416.0, 416.8, and 416.9) but also received the pivotal examinations of PH, including cardiac echocardiogram or right heart catheterization. Cox proportional hazard regression analysis was also used to calculate the adjusted HR for mortality. The results will be compared with our main results to verify whether there were any significant differences between the overall PH patients and among them who received examinations for PH. Second, we reviewed the charts for all patients with an ICD‐9‐CM diagnosis code of PH (416.0, 416.8, and 416.9) from 2005 to 2014 at Chi‐Mei Medical Center (Tainan, Taiwan). Our goal was to understand whether the 416.0 code had been used correctly. A PH specialist reviewed patient discharge records, and any prior work‐up of PH was noted. In addition to examining the accuracy of the diagnosis, the reviewer also assigned the patients to various classifications according to World Health Organization (WHO) classification in 2008. According to this result, we can further investigate the sensitivity, specificity, and predictive values of ICD coding compared with the clinical diagnosis.

## Results

### Characteristics of the Study Population

From January 1999 to December 2011, a total of 1092 newly diagnosed PH patients were identified. We also enrolled 8736 age, sex, and chronic disease–matched patients without PH for comparison. All patients were tracked from the index date to identify which patients subsequently died. The mean age of the patients with PH was 58.95±23.34 years, the majority were male (56.14%), and most did not have chronic diseases such as hypertension, diabetes, hyperlipidemia, or CAD. The detailed characteristics of both cohorts are provided in Table 1. In addition, the characteristics of PH patients before matching are presented in Table 2, while it does not show specific differences compared with the ones postmatching. The most common etiology of PH was COPD (50.37%), followed by idiopathic PH (17.31%), CTD (16.76%), CHD (11.81%), and pulmonary embolism (3.75%). Except for the patients with an etiology of CHD and idiopathic PH, most of the patients were elderly. In contrast to the similar distribution of sex in the patients with an etiology of COPD and pulmonary embolism, the patients with an etiology of CHD, CTD, and idiopathic PH were predominantly female (63.57%, 68.85%, and 61.38%, respectively, *P*<0.0001) (Table 3). Most of these patients were free from chronic diseases.

**Table 1:** Baseline Characteristics of the PH Patients and the Matched Controls

Characteristic	PH	Controls	*P‐*Value^a^
	n=1092	n=8736	
	n (%)	n (%)	
Age, y			
<50	320 (29.30)	2412 (27.61)	0.4994
50 to 64	217 (19.87)	1775 (20.32)	
≥65	555 (50.82)	4549 (52.07)	
Age, mean±SD	58.95±23.34^b^	59.55±23.03^b^	0.4226
Sex			
Female	479 (43.86)	3763 (43.07)	0.6193
Male	613 (56.14)	4973 (56.93)	
Hypertension			
Yes	431 (39.47)	3633 (41.59)	0.1803
No	661 (60.53)	5103 (58.41)	
Diabetes mellitus			
Yes	197 (18.04)	1627 (18.62)	0.6399
No	895 (81.60)	7109 (81.38)	
Hyperlipidemia			
Yes	84 (7.69)	645 (7.38)	0.7133
No	1008 (92.31)	8091 (92.62)	
CAD			
Yes	230 (21.06)	1715 (19.63)	0.2632
No	862 (78.94)	7021 (80.37)	

CAD indicates coronary artery disease; PH, pulmonary hypertension.

a *P*‐values were calculated based on the Pearson's χ^2^ test.

b Data are mean±SD.

**Table 2:** Baseline Characteristics of the PH Patients Before Matching

Characteristic	PH
	n=1112
	n (%)
Age, y	
<50	325 (29.32)
50 to 64	221 (19.87)
≥65	566 (50.90)
Age, mean±SD	59.18±23.34^a^
Sex	
Female	490 (44.06)
Male	622 (55.94)
Hypertension	
Yes	451 (40.56)
No	661 (59.44)
Diabetes mellitus	
Yes	212 (19.06)
No	900 (80.94)
Hyperlipidemia	
Yes	85 (7.64)
No	1027 (92.36)
CAD	
Yes	250 (22.48)
No	862 (77.52)

CAD indicates coronary artery disease; PH, pulmonary hypertension.

a Data are mean±SD.

**Table 3:** Incidence Densities of Death Among Patients With Different Etiologies of PH by Sex and Age

Characteristic	Pulmonary Embolism	CTDs	CHD	COPD	Idiopathic	*P‐*Value^a^
Total=1092 (100%)	n=41 (3.75%)	n=183 (16.76%)	n=129 (11.81%)	n=550 (50.37%)	n=189 (17.31%)	
Age, y						
<50	8 (19.51)	43 (23.50)	107 (82.95)	72 (13.09)	90 (47.62)	<0.0001
50 to 64	13 (31.71)	43 (23.50)	15 (11.63)	101 (18.36)	45 (23.81)	
≥65	20 (48.78)	97 (53.01)	7 (5.43)	377 (68.55)	54 (28.57)	
Age, mean±SD	63.14±17.16	62.80±18.62	27.11±22.07	67.56±18.07	21.02±22.02	<0.0001
Sex						
Female	23 (56.10)	126 (68.85)	82 (63.57)	266 (48.36)	116 (61.38)	<0.0001
Male	18 (43.90)	57 (31.15)	47 (36.43)	284 (51.64)	73 (38.62)	
Hypertension						
Yes	14 (34.15)	83 (45.36)	13 (10.08)	257 (46.73)	64 (33.86)	<0.0001
No	27 (65.85)	100 (54.64)	116 (89.92)	293 (53.27)	125 (66.14)	
Diabetes mellitus						
Yes	9 (21.95)	27 (14.75)	5 (3.88)	116 (21.09)	40 (21.16)	<0.0001
No	32 (78.05)	156 (85.25)	124 (96.12)	434 (78.91)	149 (78.84)	
Hyperlipidemia						
Yes	6 (14.63)	12 (6.56)	2 (1.55)	50 (9.09)	14 (7.41)	0.0214
No	35 (85.37)	171 (93.44)	127 (98.45)	500 (90.91)	175 (92.59)	
CAD						
Yes	11 (26.83)	40 (21.86)	7 (5.43)	138 (25.09)	34 (17.99)	<0.0001
No	30 (73.17)	143 (78.14)	122 (94.57)	412 (74.91)	155 (82.01)	

CAD indicates coronary artery disease; CHD, congenital heart disease; COPD, chronic obstructive pulmonary disease; CTDs, connective tissue diseases; PH, pulmonary hypertension.

a *P*‐values were calculated based on the χ^2^ test.

### Long‐Term Risk of Mortality

In the 12‐year follow‐up period, the patients with PH had a higher mortality rate compared with the age‐, sex‐, and chronic diseases matched–control group (56.45 versus 18.51/1000 person‐years, *P*<0.0001) (Table 4). Among the patients with PH, the mortality rate was higher in the older (<50 years versus ≥65 years, 92.09 versus 15.11/1000 person‐years) and male patients. However, after stratifying by age and sex, the younger (<50 years) and female patients had a higher risk of mortality (IRR: 12.74, 95% CI: 7.45–21.78 and 3.47, 95% CI: 2.90–4.15, respectively, *P*<0.0001). By further classifying the patients by age and sex, both the elderly male and female patients had a higher mortality rate (84.49 and 99.05/1000 person‐years, *P*<0.0001). However, compared with the controls, the younger patients had a higher risk of death, and especially the women (IRR: 16.32, 95% CI: 8.12–32.8, *P*<0.0001) (Table 5). In addition, PH also resulted in a negative impact on survival in the patients with chronic diseases. Compared with the control group, once PH developed in the patients with hypertension (IRR: 2.57, 95% CI: 2.17–3.04), diabetes (IRR: 2.21, 95% CI: 1.72–2.83), hyperlipidemia (IRR: 2.78, 95% CI: 1.67–4.63), or CAD (IRR: 2.22, 95% CI: 1.76–2.8) (all *P*<0.0001), the risk of death increased.

**Table 4:** Incidence Densities of Death Between the PH Patients and the Matched Controls

Characteristic	PH				Control				IRR (95% CI)	*P‐*Value^b^
	N	Death, n (%)	PY	Rate^a^	N	Death, n (%)	PY	Rate		
All	1092	339 (56.45)	6005.42	56.45	8736	1083 (18.51)	58 507.56	18.51	3.04 (2.69–3.44)	<0.0001
Age, y										
<50	320	34 (10.63)	2250.86	15.11	2412	22 (0.91)	18 558.53	1.19	12.74 (7.45–21.78)	<0.0001
50 to 64	217	63 (29.03)	1126.75	55.91	1775	89 (5.01)	11 551.37	7.7	7.26 (5.26–10.02)	<0.0001
≥65	555	242 (43.60)	2627.81	92.09	4549	972 (21.37)	28 397.65	34.23	2.68 (2.33–3.09)	<0.0001
Sex										
Male	479	175 (36.53)	2610.99	67.02	3763	627 (16.66)	25 568.21	24.52	2.73 (2.31–3.23)	<0.0001
Female	613	164 (26.75)	3394.43	48.31	4973	456 (9.17)	32 939.35	13.84	3.47 (2.90–4.15)	<0.0001
Comorbidity										
Hypertension	431	166 (38.52)	2042.64	81.27	3633	687 (18.91)	21 705.43	31.65	2.57 (2.17–3.04)	<0.0001
Diabetes mellitus	197	75 (38.07)	935.64	80.16	1627	367 (22.56)	10 105.88	36.32	2.21 (1.72–2.83)	<0.0001
Hyperlipidemia	84	19 (31.75)	340.58	55.79	645	67 (10.39)	3342.9	20.04	2.78 (1.67–4.63)	<0.0001
CAD	230	89 (38.70)	1152.04	77.25	1715	361 (21.05)	10 358.37	34.85	2.22 (1.76–2.80)	<0.0001
Follow‐up										
0 to 6 months	1092	95 (8.70)	507.40	187.23	8736	71 (0.81)	4278.16	16.60	11.28 (8.30–15.34)	<0.0001
6 to 12 months	960	35 (3.65)	462.19	75.73	8355	65 (0.78)	4092.34	15.88	4.77 (3.16–7.19)	<0.0001
1 to 2 years	890	49 (5.50)	827.24	59.23	7990	159 (1.99)	7593.58	20.94	2.83 (2.05–3.90)	<0.0001
≥2 years	772	160 (20.73)	4208.59	38.02	7217	788 (10.92)	42 543.47	18.52	2.05 (1.73–2.43)	<0.0001

CAD indicates coronary artery disease; IRR, incidence rate ratio; PH, pulmonary hypertension; PY, person‐years.

a Rate per 1000 person‐years.

b *P*‐values were calculated based on the χ^2^ test.

**Table 5:** Incidence Densities of Death Between PH Patients and Controls by Sex and Age

Characteristic	PH Group				Controls				IRR (95% CI)	*P‐*Value^b^
	N	Death, n (%)	PY	Rate^a^	N	Death, n (%)	PY	Rate		
Female										
All	613	164 (26.75)	3394.43	48.31	4973	456 (9.17)	32 939.35	13.84	3.47 (2.9–4.15)	<0.0001
Age, y										
<50	205	23 (11.22)	1411.63	16.29	1581	12 (0.76)	12 020.11	1.00	16.32 (8.12–32.8)	<0.0001
50 to 64	133	35 (26.32)	728.10	48.07	1142	50 (4.38)	7601.50	6.58	7.31 (4.74–11.26)	<0.0001
≥65	275	106 (38.56)	1254.69	84.49	2250	394 (17.51)	13 317.74	29.58	2.83 (2.28–3.51)	<0.0001
Male										
All	479	175 (36.53)	2610.99	67.02	3763	627 (16.66)	25 568.21	24.52	2.73 (2.31–3.23)	<0.0001
Age, y										
<50	115	11 (9.57)	839.23	13.11	831	10 (1.20)	6538.43	1.53	8.57 (3.64–20.18)	<0.0001
50 to 64	84	28 (33.33)	398.64	70.24	633	39 (6.16)	3949.87	9.87	7.11 (4.38–11.56)	<0.0001
≥65	280	136 (48.57)	1373.12	99.05	2299	578 (25.14)	15 079.91	38.33	2.58 (2.14–3.11)	<0.0001

IRR indicates incidence rate ratio; PH, pulmonary hypertension; PY, person‐years.

a Rate per 1000 person‐years.

b *P*‐values were calculated based on the χ^2^ test.

**Figure 1:**
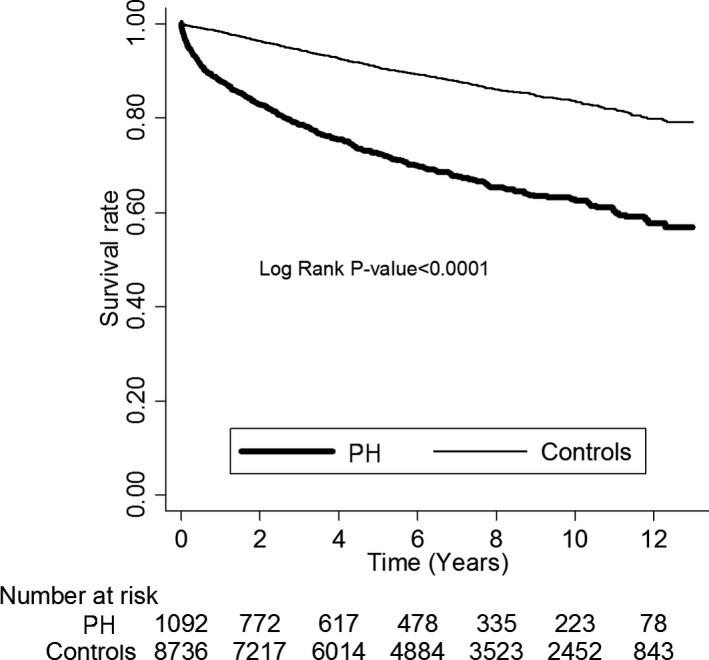
Kaplan–Meier estimates of 12‐year survival between PH patients and the matched controls. PH indicates pulmonary hypertension.

The estimated survival of the PH patients declined significantly with time. At 1, 5, and 10 years, the survival rates were 87.9%, 72.5%, and 62.6%, respectively, compared with 98.4%, 90.8%, and 83.6% in the control group (Figure 1). The adjusted HR of the PH patients was 3.3‐fold higher (adjusted HR: 3.3, 95% CI: 2.92–3.73, *P*<0.001) compared with the controls. Notably, most of the PH patients died within the first 6 months after being diagnosed (IRR: 11.28, 95% CI: 8.3–15.34, *P*<0.0001). Subsequently, the risk of mortality declined with time (6–12 months: IRR: 4.77, 95% CI: 3.16–7.19, *P*<0.0001). Of the patients who died of PH, most were older males (adjusted HR: 10.77, 95% CI: 8.17–14.22 and 1.34, 95% CI: 1.20–1.49, respectively, *P*<0.05) (Table 6). Interestingly, the PH patients with chronic diseases including hypertension (adjusted HR: 1.40, 95% CI: 1.25–1.56), diabetes mellitus (adjusted HR: 1.49, 95% CI: 1.32–1.67), and CAD (adjusted HR: 1.14, 95% CI: 1.01–1.28) (all *P*<0.05) were at a higher risk of mortality.

**Table 6:** Crude and Adjusted Hazard Ratios^a^ for Death From PH Among the Study Cohort During the Follow‐Up Period

Cohort All (n=9783)	Crude Hazard Ratio (95% CI)	Adjusted Hazard Ratio (95% CI)
PH		
Yes	3.01^b^ (2.67–3.41)	3.30^b^ (2.92–3.73)
No (reference)	1.000	1.000
Age, y		
<50 (reference)	1.000	1.000
50 to 64	4.41^b^ (3.24–5.99)	3.73^b^ (2.73–5.09)
≥65	14.36^b^ (10.99–18.77)	10.77^b^ (8.17–14.22)
Sex		
Male	1.67^b^ (1.51–1.86)	1.34^b^ (1.20–1.49)
Female (reference)	1.000	1.000
Hypertension		
Yes	2.54^b^ (2.28–2.82)	1.40^b^ (1.25–1.56)
No (reference)	1.000	1.000
Diabetes mellitus		
Yes	2.17^b^ (1.94–2.43)	1.49^b^ (1.32–1.67)
No (reference)	1.000	1.000
Hyperlipidemia		
Yes	1.04 (0.83–1.29)	0.70^b^ (0.56–0.88)
No (reference)	1.000	1.000
CAD		
Yes	2.11^b^ (1.89–2.36)	1.14^b^ (1.01–1.28)
No (reference)	1.000	1.000

CAD indicates coronary artery disease; PH, pulmonary hypertension.

a Model was adjusted by age, sex, hypertension, diabetes mellitus, hyperlipidemia, and CAD.

b *P*<0.05.

### Survival by Etiology of PH

**Figure 2:**
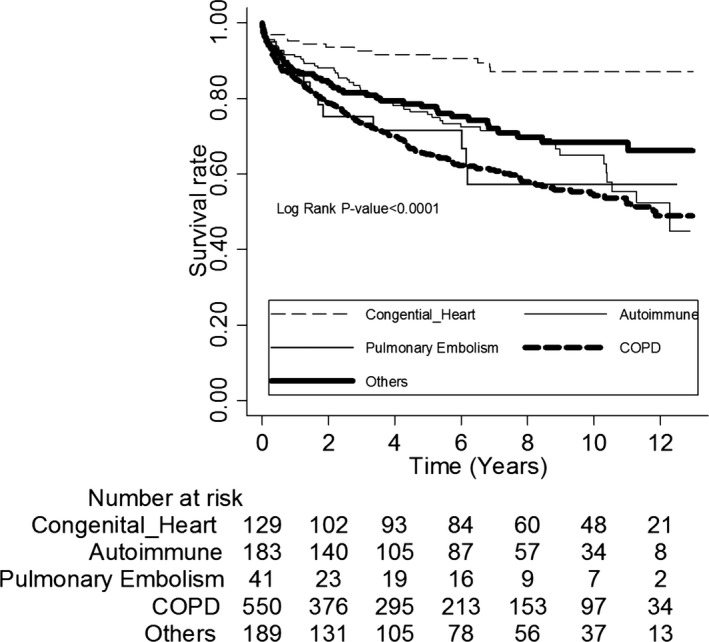
Kaplan–Meier estimates of 12‐year survival among patients with different etiologies of PH. CHD, congenital heart disease; CTDs, connective tissue diseases; COPD, chronic obstructive pulmonary disease; PH, pulmonary hypertension.

Among the different etiologies of PH, the patients with COPD (adjusted HR: 3.2, 95% CI: 2.76–3.71), idiopathic PH (adjusted HR: 4.22, 95% CI: 3.15–5.67), and pulmonary embolism (adjusted HR: 4.64, 95% CI: 2.74–7.87, all *P*<0.05) were at a higher risk of mortality compared to those with CTDs (adjusted HR: 2.76, 95% CI: 2.10–3.62, *P*<0.05) (Table 7). Notably, after adjusting for age, sex, and chronic diseases, the HR of CHD increased significantly from 0.82 (95% CI: 0.48–1.39) to 4.45 (95% CI: 2.58–7.69). The main reason was that most patients with CHD‐associated PH were younger (64.29% <50 years, *P*<0.0001) compared to those with other etiologies of PH. Thus, the age‐adjusted HR failed to fairly reflect the significance of congenital heart disease on mortality. The survival rates of the patients with pulmonary embolism, COPD, and idiopathic PH at 1 and 10 years were 87.35%, 71.58%, and 57.27% versus 87.23%, 77.77%, and 68.41%, respectively (*P*<0.0001), which were significantly lower compared with the survival rates in the PH patients overall (Figure 2). In contrast, CHD‐associated PH resulted in better outcomes, and the estimated survival rates at 1, 5, and 10 years were 95.3%, 91.64% and 87.09%. Notably, except for CHD‐induced PH, most of the patients with other etiologies of PH died at an age above 65 years (Table 8). There were no significant differences between sexes in subgroup analysis. Regarding repeated hospitalization, no significant differences were observed among those with different etiologies of PH (Table 9).

**Table 7:** Crude and Adjusted Hazard Ratios of Mortality Among Different Etiologies of PH

	N	Death	PY	Incidence^a^	Crude HR (95% CI)	Adjusted HR^b^ (95% CI)
PH group						
Pulmonary embolism	41	14	189.13	74.02	3.91^c^ (2.31–6.62)	4.64^c^ (2.74–7.87)
CTDs	183	55	1030.41	53.38	2.85^c^ (2.18–3.74)	2.76^c^ (2.10–3.62)
CHD	129	14	931.90	15.02	0.82 (0.48–1.39)	4.45^c^ (2.58–7.69)
COPD	550	209	2841.98	73.54	3.91^c^ (3.37–4.53)	3.20^c^ (2.76–3.71)
Idiopathic	189	47	1012.00	46.44	2.48^c^ (1.85–3.31)	4.22^c^ (3.15–5.67)
Controls	8736	1083	58 507.56	18.51	1.000	1.000

CHD indicates congenital heart disease; COPD, chronic obstructive pulmonary disease; CTDs, connective tissue diseases; HR, hazard ratio; PH, pulmonary hypertension; PY, person‐years.

a Per 1000 person‐years.

b Model was adjusted for age, sex, hypertension, diabetes mellitus, hyperlipidemia, and coronary artery disease.

c *P*<0.05.

**Table 8:** Age and Sex Distribution of Mortality Among the Different Etiologies of PH

Characteristic	Pulmonary Embolism	CTDs	CHD	COPD	Idiopathic	*P‐*Value
Total=339 (100%)	n=14 (4.13%)	n=55 (16.22%)	n=14 (4.13%)	n=209 (61.65%)	n=47 (13.86%)	
Age, y						<0.0001
<50	1 (7.14)	7 (12.73)	9 (64.29)	8 (3.83)	9 (19.15)	
50 to 64	5 (35.71)	6 (10.91)	2 (14.29)	37 (17.70)	13 (27.66)	
≥65	8 (57.14)	42 (76.36)	3 (21.43)	164 (78.47)	25 (53.19)	
Age, mean±SD	69.29±12.58	71.62±14.88	35.45±26.19	72.55±12.19	64.42±16.86	<0.0001
Sex						0.4112
Female	8 (57.14)	29 (52.73)	9 (64.29)	93 (44.50)	25 (53.19)	
Male	6 (42.86)	26 (47.27)	5 (35.71)	116 (55.50)	22 (46.81)	

CHD indicates congenital heart disease; COPD, chronic obstructive pulmonary disease; CTDs, connective tissue diseases; PH, pulmonary hypertension.

**Table 9:** Crude and Adjusted Hazard Ratios of Repeated Hospitalization Among Different Etiologies‐Induced PH Patients

	N	Repeated Hospitalization	Crude OR (95% CI)	Adjusted OR^a^ (95% CI)
PH group				
Pulmonary embolism	41	6	1.24 (0.47–3.26)	1.18 (0.44–3.14)
Autoimmune	183	29	1.36 (0.75–2.45)	1.28 (0.7–2.35)
Congenital heart disease	129	19	1.25 (0.65–2.4)	1.38 (0.7–2.72)
COPD	550	96	1.53 (0.94–2.49)	1.37 (0.82–2.3)
Others	189	23	1.000	1.000

COPD indicates chronic obstructive pulmonary disease; OR, odds ratio; PH, pulmonary hypertension.

a Model was adjusted by age, sex, hypertension, diabetes mellitus, hyperlipidemia, and coronary artery disease.

### Validation of the Accuracy of PH Diagnosis

After redefining the PH patients, there were 960 patients concomitantly receiving cardiac echocardiography and 158 patients receiving right heart catheterization (Table 10). Compared with the corresponding controls, PH patients with cardiac echocardiography presented significantly higher risk of mortality (adjusted HR: 3.53, 95% CI: 3.1–4.01, *P*<0.001). Likewise, PH patients with right heart catheterization presented 4.55‐fold higher risk of mortality (95% CI: 3.06–6.77, *P*<0.001). The results were similar to the ICD‐9‐CM‐defined PH patients that we initially recruited.

**Table 10:** Crude and Adjusted Hazard Ratios^a^ for Death From Patients With ICD‐9‐Defined PH, PH With Cardiac Echo, and PH With Cardiac Echo and Right Heart Catheterization

Cohort All (n=9783)	Crude Hazard Ratio (95% CI)	Adjusted Hazard Ratio (95% CI)
PH (n=1092)	3.01^b^ (2.67–3.41)	3.30^b^ (2.92–3.73)
Corresponded Non_PH (reference)	1.000	1.000
PH with cardiac echo (N=960)	3.19^b^ (2.81–3.62)	3.53^b^ (3.10–4.01)
Corresponded Non_PH (reference)	1.000	1.000
PH with cardiac echo and right heart catheterization (N=158)	3.64^b^ (2.47–5.38)	4.55^b^ (3.06–6.77)
Corresponded Non_PH (reference)	1.000	1.000

CAD, coronary artery disease; ICD‐9, International Classification of Diseases, Ninth Revision; PH, pulmonary hypertension.

a Model was adjusted by age, sex, hypertension, diabetes mellitus, hyperlipidemia, and CAD.

b *P*<0.05.

Furthermore, from 2005 to 2014, 216 patients were reported as having PH at discharge, including 18 coded as idiopathic pulmonary arterial hypertension (416.0), 177 coded as other chronic pulmonary heart disease (416.8), and 23 coded as unspecified pulmonary heart disease (416.9) (Table 11). Chart reviews of the patients assigned by ICD‐9‐CM codes for PH revealed that only relatively small numbers of patients were incorrectly or unclearly diagnosed (n=11, 5%) and the positive predictive value was up to 94.9%. Among the various etiologies of PH, most of the patients were defined as left heart disease–induced PH (n=122, 56.5%). Taking idiopathic pulmonary arterial hypertension as an example, the specificity of the accurate diagnosis was measured as 96.7% according to ICD coding. Nevertheless, the sensitivity was relatively lower at 63.1%. Generally speaking, both of the validation methods indicated satisfactory accuracy and reliability of the PH coding in the NHI database.

**Table 11:** PH ICD‐9 Codes Versus Chart Review Diagnosis

Code	N	Expert‐Reviewer Diagnosis	N
416.0 Idiopathic PAH	18	Unclear or not PH	3
		Class 1. PAH	12
		Class 2. PH with left heart disease	2
		Class 3. PH with lung disease	1
		Class 4. PH with chronic thromboembolism	0
		Class 5. Miscellaneous	0
416.8 Other chronic pulmonary heart disease	177	Unclear or not PH	6
		Class 1. PAH	6
		Class 2. PH with left heart disease	108
		Class 3. PH with lung disease	36
		Class 4. PH with chronic thromboembolism	17
		Class 5. Miscellaneous	2
416.9 Pulmonary heart disease unspecified	23	Unclear or not PH	2
		Class 1. PAH	1
		Class 2. PH with left heart disease	12
		Class 3. PH with lung disease	5
		Class 4. PH with chronic thromboembolism	3
		Class 5. Miscellaneous	0

ICD‐9 indicates International Classification of Diseases, Ninth Revision; PAH, pulmonary arterial hypertension; PH, pulmonary hypertension.

## Discussion

The main findings of the present study are that (1) compared with the matched cohort without PH, the mortality rate was up to 3 times higher in the patients with PH; (2) mortality was associated with male sex, older age, and concomitant comorbidities; however, a younger age and female sex were more significantly associated with mortality compared to the control group; and (3) COPD, idiopathic, and pulmonary embolism etiologies of PH contributed to a higher risk of mortality. To the best of our knowledge, this nationwide population‐based study is the first to comprehensively describe the impact of PH on mortality compared with matched controls without PH.

PH is a rare but serious progressive disease,^1^ and most current knowledge comes from small‐scale or specialized registries.^7^, ^15^, ^16^ Because the characteristics of patients with PH varies, these registries cannot reflect real‐world conditions. In addition, most previous studies have only included PH patients of specific ethnicity. Among them, the REVEAL registry was a multicentered, well‐designed study, which focused on middle‐aged white women, the majority of whom were diagnosed with idiopathic PH (46.5%), followed by CHD (11.8%) and CTD (23.9%; 13.5% scleroderma).^17^ However, none of the patients had lung disease (WHO group 3) or left heart failure (WHO group 2). The authors concluded that older males with portal hypertension or connective tissue disease–induced PH had the worst prognosis. In another study focusing on African Americans, despite the similarity in age (56.1±12.6 years) and female predominance (67.5%) of the studied population, PH was more associated with obesity, diabetes mellitus, and obstructive or restrictive spirometry patterns.^3^ In addition, Pugh and colleagues reported that WHO groups 2 and 3 were most frequently diagnosed (28% and 17%, respectively) in elderly patients (>65 years) with PH.^18^ Therefore, when looking at any registry, differences need to be identified between the target population, the intended population, and the population actually studied. Despite some recently released studies, only limited information is available regarding the prevalence of PH in Asia.^7^, ^19^, ^20^ In our longitudinal and nationwide study, we focused on a Taiwanese population. Their mean age was 58.95±23.34 years and the sex distribution was similar (males 56.14% versus females 43.86%). Most of them were free from chronic diseases, including hypertension, diabetes mellitus, and CAD. The most common etiologies contributing to PH were COPD (50.37%), followed by idiopathic PH (17.31%), CTDs (16.76%), CHD (11.81%), and pulmonary embolism (3.75%), which reflects the disease distribution in clinical practice.

Previous studies have reported that elderly male PH patients are at the highest risk of mortality.^15^, ^16^ Choudhary and colleagues reported that the prevalence of PH in African Americans above 65 years of age was 10 times that of those younger than 45 years of age.^3^ The higher prevalence of PH in the elderly may be related to decreasing compliance of the pulmonary arteries with age.^15^, ^18^ In addition, older male patients often have concomitant diseases, thereby resulting in worse outcomes.^18^ Similarly, in our study mortality was associated with male sex, older age, and concomitant comorbidities. However, as we compared PH patients with sex‐ and age‐matched controls, younger and female patients presented a higher risk of mortality. Regarding the differences in survival between sexes, emerging data suggest that estrogens may interact with local receptors to enhance potential damage on the pulmonary vascular system.^8^, ^21^, ^22^ Therefore, most subtypes of PH are characterized by a greater susceptibility to disease among females, but previous studies also indicated better survival in females while this “estrogen paradox” remains a mystery.^21^, ^22^ Also, the effect of sex hormones is not present in all kinds of PH.^22^ In contrast to the similar distribution of sex in COPD and pulmonary embolism–induced PH, CHD and autoimmunity were more common in the female patients.^21^, ^22^ In our opinion, the exact role of sex in survival from PH remains to be elucidated. Regarding our finding of higher mortality of younger PH patients, PH is frequently unrecognized in childhood because of a lack of clinical signs, while those who are diagnosed at an early stage may present fulminate symptoms resulting in worse survival compared with the age‐matched normal population.^23^ Notably, with the advancement of PH therapies, long‐term follow‐up is crucial to investigate the changes of survival in PH patients with various characteristics.

Among the different etiologies of PH, the survival rates varied. In previous studies, the survival rates of patients with CTD have been inferior to those of patients with idiopathic PH.^6^ Zhang et al reported 1‐ and 3‐year survival estimates of 92.1% and 75.1% in patients with idiopathic PH compared to 85.4% and 53.6% in patients with CTD‐induced PH.^6^ In other studies, significantly lower PH‐associated mortality rates have been reported for those with pulmonary embolism and emphysema.^2^ Compared with congenital and idiopathic PH, systemic sclerosis and human immunodeficiency virus‐induced PH have been reported to have worse survival rates.^2^ However, a comprehensive and large‐scale study to investigate the outcomes with regard to different etiologies of PH is still required. In our study, the patients with COPD, idiopathic, and pulmonary embolism had a higher risk of mortality (adjusted HR: 3.2, 95% CI: 2.76–3.71; 4.22, 95% CI: 3.15–5.67; and 4.64, 95% CI: 2.74–7.87, respectively, *P*<0.05), compared with CTD and CHD‐induced PH (Table 4). In contrast to other studies, 65.9% of our PH patients had an etiology of COPD, especially the older patients. The development of PH implies that the severity of pulmonary disease has progressed. A previous study reported that multiple sclerosis resulted in worse outcomes.^24^ In this study, the PH patients with an etiology of CTD had a higher prevalence of systemic lupus erythematosus and better survival. Consistent with previous studies, we found that CHD‐associated PH predicted a better prognosis, with the patients surviving for decades if managed properly.^2^ The major reason may be the preservation of right ventricular function, as in CHD the right ventricle is exposed to higher pressures at the early stage, which leads to better adaptive properties.^25^

In this study, the PH patients were at the highest risk of mortality within the first 6 months after the diagnosis (IRR: 11.28, 95% CI: 8.3–15.34, *P*<0.0001). This may be because of the underdiagnosis of PH in these patients, where most of the subtle symptoms at the early stage may be ignored until the disease exacerbates. Subsequently, after the effects of PH therapy, the risk of mortality declined (6–12 months: IRR: 4.77, 95% CI: 3.16–7.19, *P*<0.0001). Furthermore, in our study, the estimated overall survival of the PH patients was higher than in previous studies. At 1 and 5 years, the survival rates were 87.9% and 72.5% compared with 68% and 20.8% reported by Benza et al.^15^ This may be because of the heterogeneity of the population or the availability of medicine after the initiation of the NHI program in Taiwan.

In contrast to previous studies reporting a high incidence of concomitant chronic diseases,^2^, ^5^ the incidence was lower in our study including 39.46% with hypertension, 18.04% with diabetes mellitus, 7.69% with hyperlipidemia, and 21.06% with CAD. However, these chronic diseases did have a negative impact on survivals in the patients with PH. Compared with the control group, the patients with both PH and chronic diseases had a more than 2‐fold higher risk of mortality. This highlights the importance of targeting these risk factors for the prevention and therapy of PH.

This study has several strengths. First, it involved an unselected nationwide population with a large number of patients with PH. By recruiting 1092 patients during the 12‐year study period, this study provided adequate statistical power for the analysis of PH‐related long‐term outcomes. Second, we compared study subjects with matched control subjects in comparison with a general population. In addition, this study included patients with various kinds of PH, including idiopathic PH, CTDs, CHD (WHO class 1), COPD (WHO group 3), and thromboembolic‐induced PH (WHO group 4), which allowed for a comprehensive investigation of the effects of various etiologies of PH.

There are several limitations to this study. First, the miscode of PH is possible. Nevertheless, to overcome the inherent limitations of observational studies based on administrative claims data, we verified the accuracy of PH diagnosis by 2 validation methods including redefining the diagnosis by cardiac echocardiogram or right heart catheterization and chart review of the specialists. Also, we conducted a matched analysis, allowing us to reduce the effects of confounding when using observational data. Second, the claims data did not include left heart or portal hypertension–induced PH, which are 2 pivotal types of PH. However, all of the patients that we recruited had PH with low wedge pressure. Third, information on medications and functional study results, such as the 6‐minute walk test, were lacking. Finally, without longitudinal follow‐up data of right heart pressure as measured by echocardiography or Swan‐Ganz catheter, the results may not reflect the severity of disease. Repeated hospitalization and specific prescriptions of PH target therapies help differentiate the severity, but the accuracy and prevalence may be influenced by the health insurance system.

## Conclusions

PH remains a fatal disease in Taiwan with a 3.3‐fold higher risk of mortality. Compared with the matched controls, PH‐induced mortality was associated with female sex, younger age, and the coexistence of chronic diseases. Among the different etiologies of PH, COPD and pulmonary embolism contributed most to the increased risk of mortality. These findings should alert clinicians to the importance of detecting the development of PH at an early stage and also to treat combined chronic diseases. In addition to focusing on older patients, younger female patients may also be at a high risk and require an early and appropriate diagnosis.

## Sources of Funding

We received research program support from Chi‐Mei Medical Center.

## Disclosures

None.
